# Analytical Validation of a Multielement ICP-MS Method for Human Serum and Its Application to Trace Element Biomonitoring in Hemodialysis Patients

**DOI:** 10.3390/toxics14100884

**Published:** 2026-10-01

**Authors:** Sara Valderrama Sanz, Raquel Sánchez Romero, Ricardo Molina Gasset, José Luis Todolí Torró

**Affiliations:** 1 Clinical Analysis Service, Hospital Virgen de los Lirios, Polígono de Caramanchel s/n, 03804 Alcoi, Spain; saravalderrama@hotmail.com (S.V.S.); molina_ric@gva.es (R.M.G.); 2 Analytical Chemistry Department, Nutrition and Food Sciences, University of Alicante, Carretera San Vicente del Raspeig s/n, 03690 San Vicente del Raspeig, Spain; r.sanchez@ua.es

**Keywords:** ICP–MS, human serum, trace elements, analytical validation, biomonitoring, measurement uncertainty

## Abstract

Reliable determination of trace elements in human serum is essential for clinical assessment, toxicological investigations and human biomonitoring. This study describes the development and analytical validation of a multielement inductively coupled plasma mass spectrometry (ICP–MS) method for the simultaneous determination of essential and potentially toxic trace elements in human serum (Ti, V, Cr, Mn, Co, Ni, Cu, Zn, As, Cd, Ba, Bi) using a simple direct-dilution procedure with 1% (*v*/*v*) nitric acid. Method validation included assessment of linearity, limits of detection and quantification, carry-over, precision, trueness, measurement uncertainty and analyte stability. Calibration curves showed excellent linearity, with coefficients of determination (R^2^) ranging from 0.9993 to 1.0000 across analyte-specific calibration ranges of 0–10, 0–50, and 0–500 µg L^−1^. Repeatability ranged from 0.69% (Cu) to 14.5% (Ba), while intermediate precision ranged from 1.32% (Zn) to 81.1% (Cd and Bi at low concentrations). Recoveries obtained using certified serum reference materials ranged from 55% (V) to 206% (Bi) across the evaluated conditions. Meanwhile, limits of detection ranged from 0.01 (V) to 0.16 (Cu) µg L^−1^. Measurement uncertainty was estimated following the EURACHEM/CITAC and GUM recommendations, identifying repeatability as the principal contributor to the overall uncertainty budget. The method was successfully applied to serum samples from healthy individuals and patients undergoing chronic hemodialysis. The proposed methodology combines simple sample preparation with comprehensive metrological characterisation, providing practical approach multielement serum analysis and its application to trace-element biomonitoring investigations.

## 1. Introduction

Trace elements play essential roles in numerous physiological processes, acting as structural components of proteins, enzyme cofactors, signaling mediators, and regulators of oxidative balance. However, disturbances in trace element homeostasis may contribute to the development and progression of several pathological conditions. While deficiencies or altered homeostasis of essential elements such as zinc (Zn), manganese (Mn), and copper (Cu) may be associated with impaired physiological and antioxidant functions, excessive exposure to potentially toxic elements including arsenic (As), chromium (Cr), cobalt (Co) and nickel (Ni) may be associated with oxidative stress, inflammation, and cellular toxicity [1,2,3,4].

Beyond their effects on systemic homeostasis, exposure to toxic trace elements and heavy metals may also have broader biological consequences. Experimental evidence has shown that heavy metals can adversely affect reproductive processes, including gonadal integrity and sperm DNA stability, with combined metal exposure potentially producing greater effects than individual metals [5]. Oxidative damage is another important mechanism underlying metal toxicity. In particular, copper can participate in redox reactions that promote reactive oxygen species generation and oxidative DNA damage, providing a mechanistic link between metal exposure and cellular injury [6].

Human biomonitoring has emerged as a valuable tool for assessing both nutritional status and environmental or occupational exposure to trace elements. Among the available biological matrices, serum is widely used because it reflects the circulating fraction of many elements and can be obtained through minimally invasive procedures routinely performed in clinical practice [7,8]. Nevertheless, the determination of trace elements in serum remains analytically challenging due to the complex composition of the matrix, the low concentrations at which many analytes are present, and the risk of contamination during sample collection, storage, and preparation.

Several analytical techniques have been employed for trace element determination in biological samples, including flame atomic absorption spectrometry (FAAS), graphite furnace atomic absorption spectrometry (GFAAS), inductively coupled plasma optical emission spectrometry (ICP-OES), and inductively coupled plasma mass spectrometry (ICP-MS) [9]. Among these methodologies, ICP-MS is currently considered the reference technique for multielement biomonitoring owing to its excellent sensitivity, wide linear dynamic range, low detection limits, and ability to simultaneously quantify multiple analytes within a single analytical run [10]. Furthermore, the incorporation of collision/reaction cell technology has substantially improved analytical performance by minimizing polyatomic interferences, thereby expanding the applicability of ICP-MS to complex biological matrices [11].

Despite these advantages, the reliability of ICP-MS measurements depends on rigorous analytical validation. Biological matrices such as serum are particularly susceptible to matrix-induced signal suppression, contamination artifacts, carry-over effects, instrumental drift, and variability associated with sample preparation procedures [12]. Consequently, comprehensive validation studies addressing analytical sensitivity, precision, trueness, linearity, measurement uncertainty, and analyte stability are essential before applying ICP-MS methodologies to human biomonitoring investigations [13,14]. However, many published biomonitoring studies focus primarily on biological findings while providing limited information regarding analytical performance characteristics, thereby complicating the comparison and interpretation of results across laboratories [15].

Patients undergoing chronic hemodialysis constitute a particularly relevant population for trace element biomonitoring. In addition to the profound alterations in renal excretory function associated with end-stage kidney disease, these individuals are exposed to multiple factors capable of modifying trace element homeostasis, including dietary restrictions, chronic inflammation, medication use, dialysis membranes, and repeated exposure to large volumes of dialysis fluid [16,17,18]. As a result, both depletion of essential trace elements and accumulation of potentially toxic metals have been reported in hemodialysis populations, although findings remain heterogeneous and are often influenced by methodological differences among studies [19,20,21].

Therefore, the availability of validated analytical methodologies capable of accurately determining multiple trace elements in serum is crucial for generating reliable biomonitoring data in clinical and epidemiological investigations. In this context, the aim of the present study was to develop, optimize, and validate a multielement ICP-MS method for the determination of trace elements in human serum. The validation protocol included assessment of limits of detection and quantification, carry-over effects, precision, trueness, linearity, measurement uncertainty, and analyte stability. In addition, the applicability of the validated methodology was demonstrated through its use in a clinical biomonitoring study involving patients undergoing chronic hemodialysis and healthy controls. It is important to underline that the suitability of serum for trace-element biomonitoring is element-dependent, as the distribution of individual elements among blood compartments varies considerably. Therefore, serum should be regarded as a relevant matrix for selected elements and specific biomonitoring objectives rather than as a universally optimal matrix for all trace elements.

## 2. Materials and Methods

### 2.1. Reagents and Standards

Ultrapure water (18.2 MΩ·cm) obtained from a Milli-Q purification system (Millipore, Bedford, MA, USA) was used throughout the study. Nitric acid (HNO_3_, suprapure grade, 65%) was purchased from Merck (Darmstadt, Germany).

Multi-element calibration standards were prepared from commercially available ICP-MS stock solutions (1000 mg L^−1^, CPAchem, Stara Zagora, Bulgaria). Working calibration solutions were freshly prepared by serial dilution in 1% (*v*/*v*) HNO_3_.

Scandium (^45^Sc), germanium (^72^Ge), rhodium (^103^Rh) and rhenium (^185^Re) were employed as internal standards to compensate for instrumental drift and residual matrix effects. Internal standard solutions were prepared daily in 1% (*v*/*v*) HNO_3_. The internal standards were selected to provide mass coverage across the investigated elemental panel, with the internal standard masses distributed across the low-, intermediate-, and high-mass regions of the analytes. Internal standard solutions were prepared daily in 1% (*v*/*v*) HNO_3_ at a final concentration of 50 ng mL^−1^ (see Table 1).

For method validation, ClinChek^®^ Serum Control Level I and Level II certified reference materials (Recipe Chemicals + Instruments GmbH, Munich, Germany) were used for precision and trueness assessment.

### 2.2. Instrumentation

Trace element determinations were performed using an Agilent 7700x inductively coupled plasma mass spectrometer (ICP-MS) (Agilent Technologies, Santa Clara, CA, USA) equipped with a MicroMist concentric nebulizer, a quartz spray chamber, and an octopole collision/reaction cell.

The instrument was operated in helium collision mode at a flow rate of 4.5 mL min^−1^ to minimize polyatomic interferences. Daily optimization procedures were performed according to the manufacturer’s recommendations to maximize sensitivity and ensure oxide and doubly charged ion formation remained within acceptable limits (i.e., <2%). Helium collision mode was used as the common operating mode for the complete elemental panel to minimize polyatomic interferences under the selected conditions.

Instrument operating conditions are summarized in Table 1.

### 2.3. Serum Samples

Human serum samples were obtained from 199 participants recruited at Hospital Virgen de los Lirios (Alcoy, Spain) between 2015 and 2017, including 82 healthy controls (CNTRL) and 117 patients undergoing chronic hemodialysis (HD). The control group was selected to match the HD cohort according to age and sex. The mean age was 71.7 ± 8.8 years in the HD group and 71.2 ± 13.1 years in the control group. Among HD patients, 79 (67.5%) were male. For the healthy control group, exclusion criteria included evidence of renal impairment, defined as an estimated glomerular filtration rate below 60 mL min^−1^ 1.73 m^−2^, or active neoplastic disease.

Clinical records were reviewed to identify relevant comorbidities, including arterial hypertension, diabetes mellitus, cardiovascular disease, liver disease and osteoporosis. Lifestyle factors, including current smoking, habitual alcohol consumption, and habitual salad, meat and fish intake, were also recorded. Among HD patients, dialysis-related variables included dialysis vintage (<12 or ≥12 months), dialysis session duration (<240 or ≥240 min), hemodialysis modality (low-flux HD or online hemodiafiltration), dialyzer filter type (FX or non-FX), and dialysis water calcium concentration (1.25 or ≥1.5 mmol L^−1^). These variables were considered as potential sources of biological or treatment-related variability in serum trace-element concentrations.

Detailed information on medication and nutritional supplementation was not systematically available for all participants and was therefore not included as a covariate in the present analysis. This limitation was considered when interpreting the clinical application of the analytical method.

The study was conducted in accordance with the Declaration of Helsinki and approved by the corresponding institutional ethics committee. Written informed consent was obtained from all participants prior to inclusion.

The clinical application was primarily intended to demonstrate the applicability of the validated analytical method to real serum samples from healthy individuals and patients undergoing chronic hemodialysis, rather than to provide an independent clinical or statistical comparison between the two groups.

### 2.4. Sample Collection, Storage and Preparation

Venous blood samples were collected using trace-element certified BD Vacutainer tubes specifically designed to minimize metal contamination.

For the healthy control group, blood samples were obtained under fasting conditions following routine clinical laboratory procedures. For the haemodialysis cohort, blood samples were collected immediately before the start of the dialysis session (pre-dialysis), in order to avoid the acute influence of the dialysis procedure on serum trace element concentrations and biochemical parameters. Blood samples were collected using BD Vacutainer^®^ trace element serum tubes (REF 368380, Becton Dickinson, Franklin Lakes, NJ, USA). As serum was obtained after clot formation, no anticoagulant was used. After centrifugation, serum aliquots were transferred into acid-washed polypropylene microtubes previously decontaminated by soaking in 1% (*v*/*v*) nitric acid and rinsing with ultrapure water. Samples were stored at −80 °C until analysis. Prior to analysis, samples were thawed at room temperature and thoroughly homogenised before dilution (1:10) with ultrapure water containing 1% (*v*/*v*) ultrapure nitric acid.

During the preliminary method-development stage, three serum dilution factors (1:10, 1:20 and 1:50) were evaluated to assess their influence on analytical sensitivity, matrix effects and recovery across the investigated elemental panel. ClinChek^®^ Serum Control Level II was used as the test material. Aliquots of the control material were diluted with ultrapure water containing 1% (*v*/*v*) HNO_3_ according to the corresponding dilution factor and analysed by ICP–MS under the instrumental conditions described in Section 2.2. Recoveries were calculated by comparing the measured concentrations with the certified values of the reference material. The dilution factor providing the most appropriate overall balance between matrix reduction and analytical sensitivity across the elemental panel was selected for subsequent method validation and clinical sample analysis.

### 2.5. Analytical Panel

The elemental panel investigated in the present study was selected to encompass trace elements with different biological and toxicological relevance and substantially different concentration levels in human serum. The panel included essential elements such as Mn, Cu and Zn, elements of physiological and potentially toxicological relevance such as Ti, V, Cr, Co, Ni and Ba, and potentially toxic elements such as As, Cd and Bi. The inclusion of elements occurring at both relatively high and ultratrace concentrations was intentional, as it allowed the analytical performance of a single multielement procedure to be evaluated across a broad concentration range. This selection was also guided by the potential relevance of these elements to human biomonitoring and to populations with altered trace-element homeostasis, such as patients undergoing chronic hemodialysis. Other relevant elements, such as selenium, were not included because of the potential for significant spectral interferences under the analytical conditions used. Mercury, for instance, was excluded because of its pronounced memory effects and insufficient analytical sensitivity under the conditions evaluated. Iron was also excluded because its predominant distribution in erythrocytes makes serum less representative of total body iron status. These exclusions were therefore based on analytical and biological considerations rather than on a lack of clinical relevance of these elements.

### 2.6. Calibration Procedure

Quantification was performed by external calibration using multi-element aqueous calibration standards prepared in 1% (*v*/*v*) HNO_3_, using the same acidic matrix as that employed for sample dilution. Internal standardization was applied simultaneously by continuous online introduction of ^45^Sc, ^72^Ge, ^103^Rh, and ^185^Re to correct for instrumental signal fluctuations and residual matrix-related effects.

Calibration curves consisted of ten concentration levels covering the expected physiological and pathological concentration ranges of the investigated elements (0, 0.5, 1, 5, 10, 50, 100, 500, 1000 and 5000 μg L^−1^). The calibration range was adapted to the concentration according to the certified value for each element. Therefore, different concentration ranges were used for the individual elements, as specified in Table 2, rather than applying a single common calibration range to the complete elemental panel. In the case of real samples, the calibration range was selected by comparing the analytical responses of the elements with those for the standards.

Internal standards were continuously introduced online and used to correct instrumental drift and signal fluctuations.

Calibration linearity was assessed by least-squares regression analysis.

### 2.7. Method Validation

Method validation was carried out following IUPAC recommendations and Eurachem guidelines for analytical method validation and uncertainty estimation [13,14].

The validation strategy was designed to characterize the main analytical performance characteristics relevant to quantitative multielement determination in human serum. The method was evaluated in terms of linearity, analytical sensitivity through the limits of detection (LOD) and quantification (LOQ), carry-over, repeatability, intermediate precision, trueness, analyte stability, and measurement uncertainty. Pooled human serum was used for the assessment of precision, whereas ClinChek^®^ Serum Control Levels I and II were used for the assessment of trueness. The experimental procedures, calculation methods, and acceptance criteria applied to each validation parameter are described in the following subsections.

#### 2.7.1. Linearity

Method linearity was evaluated following the recommendations of the Eurachem [13] guideline for quantitative analytical methods.

Calibration curves were prepared using aqueous multielement standard solutions covering the analytical concentration range established for each element. Linearity was evaluated by linear regression analysis according to the recommendations of the Eurachem Guide for method validation. Residual plots were visually inspected in order to detect any systematic deviation from linearity.

#### 2.7.2. Limits of Detection and Quantification

Limits of detection (LOD) and limits of quantification (LOQ) were determined for every investigated element. Thirty reagent blanks were analysed under repeatability conditions. The standard deviation of blank measurements (σ) together with the slope of the corresponding calibration curve (m) were used to calculate detection and quantification limits according to the IUPAC recommendations.

The limit of detection was calculated as

LOD = 3σ/m(1)

where σ represents the standard deviation of blank measurements; m corresponds to the slope of the calibration curve.

The limit of quantification was calculated as

LOQ = 10σ/m(2)

For comparison with physiological serum concentrations, serum-equivalent LOQs were calculated by multiplying the LOQ obtained for the analytical solution by the final sample dilution factor (10).

#### 2.7.3. Carry-Over Assessment

Carry-over was evaluated by monitoring the response of reagent blanks following the analysis of highly concentrated solutions. Blank (i.e., 1% *v*/*v* nitric acid) measurements were performed after the introduction of trace-element solutions at concentrations of 500 and 50 µg L^−1^, using both the diluted nitric acid solution employed as the calibration matrix and a pooled human serum sample prepared according to the sample preparation procedure. The rinsing time was selected as the optimization parameter in order to maintain the established sample introduction conditions and avoid introducing additional variables that could affect the validated analytical performance. The rinsing time was increased until the analytical signal returned to the baseline. Therefore, the evolution of the blank signal was monitored during consecutive measurements, with each measurement corresponding to 1.5 min of acquisition.

The presence of carry-over was assessed by comparing the blank response obtained after the concentrated solution with the baseline blank response obtained before sample introduction.

#### 2.7.4. Precision

Precision was evaluated according to EURACHEM recommendations using pooled human serum samples. Repeatability and intermediate precision were assessed separately. Repeatability was evaluated by analysing consecutive aliquots of the pooled serum under the same analytical conditions within a single analytical sequence.

Intermediate precision was assessed by analysing the same pooled serum sample on three different analytical days over a three-month period. Nine consecutive measurements were performed on each analytical day, with an independent external calibration curve prepared for each analytical run. Internal-standard correction was applied to the analytical measurements.

For both repeatability and intermediate precision, the mean concentration and confidence interval (CI) were calculated for each element. Precision was expressed as the relative confidence interval, calculated as the ratio between the confidence interval and the corresponding mean concentration (CI/mean × 100). The resulting values were used to characterize short-term repeatability and between-day precision for the investigated elements.

#### 2.7.5. Trueness

Analytical trueness was evaluated using ClinChek^®^ Serum Control Levels I and II.

Initially, recoveries were calculated according to

Recovery (%) = [C_measured_/C_certified_] × 100 (3)

where C_measured_ represents the experimental concentration; C_certified_ corresponds to the certified value.

Although recovery provides useful information regarding analytical bias, it does not consider the uncertainties associated with the analytical measurement or the certified reference material.

Therefore, trueness was additionally evaluated according to the statistical approach proposed by Linsinger [22], based on comparison between the experimental concentration and the certified value.

The absolute analytical bias was calculated as

Δm = ∣C_m_ − C_CRM_∣ (4)

The uncertainty associated with this difference was obtained as

(5) $$u_{\Delta }=\sqrt{u_{m}^{2}+u_{CRM}^{2}}$$

where u_m_ is the standard uncertainty of the experimental measurement; u_CRM_ is the uncertainty reported for the certified reference material.

Analytical trueness was considered satisfactory whenever

Δ_m_ < 2u_Δ_ (6)

#### 2.7.6. Measurement Uncertainty

Measurement uncertainty was estimated according to the recommendations of the Eurachem/CITAC Guide Quantifying Uncertainty in Analytical Measurement and the ISO Guide to the Expression of Uncertainty in Measurement (GUM) [14]. The uncertainty budget included the three major sources of analytical variability identified during method validation: calibration, repeatability and trueness. This simplified approach was selected because these components represented the dominant contributions to the overall uncertainty of the ICP-MS measurements.

The uncertainty associated with the calibration procedure included the preparation of calibration standards, instrumental calibration and regression statistics. The relative uncertainty of calibration was calculated according to:

(7) $$\frac{u_{cal}}{x_{cal}}=\sqrt{{\left(\frac{s\left(y\right)}{y}\right)}^{2}+{\left(\frac{s\left(\bar{y}\right)}{\bar{y}}\right)}^{2}+{\left(\frac{u\left(x\right)}{x}\right)}^{2}}$$

Repeatability uncertainty was estimated from replicate measurements according to

(8) $$u_{rep}=\frac{s}{\bar{x}}$$

The uncertainty associated with trueness was calculated from the certified reference materials as

(9) $$u_{t}=100\frac{u_{\Delta }}{C_{CRM}}$$

Finally, the expanded uncertainty was obtained by combining all uncertainty components:

(10) $$U=\sqrt{u_{cal}^{2}+u_{rep}^{2}+u_{t}^{2}}$$

#### 2.7.7. Stability Study

The stability of serum trace elements was evaluated under the storage conditions routinely employed during clinical practice. Short-term stability was investigated at room temperature and under refrigerated conditions. Long-term stability was evaluated after storage at −80 °C. Concentrations obtained after storage were compared with those measured immediately after sample collection.

During the stability study, the pooled serum samples were subjected to eight freeze–thaw cycles. This study was performed using pooled human serum and was therefore not independently evaluated at multiple concentration levels.

### 2.8. Quality Assurance and Quality Control

To minimize contamination, all solutions were prepared using ultrapure water and high-purity nitric acid, and sample preparation was performed using acid-cleaned laboratory materials. Reagent blanks were analyzed every 10 samples to monitor background contamination arising from reagents and the analytical procedure. Blank responses were monitored for the target elements and considered during data evaluation.

Calibration performance was verified throughout each analytical sequence by repeated analysis of a calibration verification standard at predefined intervals. The measured concentrations were compared with the expected values to confirm the continued validity of the calibration. Calibration verification results were required to remain within the predefined acceptance criteria before the analytical sequence was continued. Certified serum control materials (ClinChek^®^ Serum Control Levels I and II) were thus included in each analytical batch and monitored every 10 analytical measurements. Clinical serum samples were analyzed in triplicate. QC results were considered acceptable when the measured concentrations were within ±20% of the certified values. These QC procedures were used to monitor analytical performance throughout each batch and to ensure the consistency of the measurements.

### 2.9. Application to Clinical Biomonitoring

Following validation, the analytical methodology was applied to serum samples from healthy controls and patients undergoing chronic hemodialysis. Under the selected operating conditions, 0.2 mL of serum were diluted to a final volume of 2 mL (1:10 dilution) using ultrapure water containing 1% (*v*/*v*) HNO_3_. As indicated in Table 1, the ICP-MS sample uptake rate was 0.4 mL/min. In the present study, 0.2 mL of serum was used because sufficient sample volume was available. However, this volume was not an intrinsic requirement of the analytical procedure. Given the direct dilution approach and the sensitivity achieved, the same 1:10 dilution could in principle be prepared using a lower serum volume (e.g., 0.05–0.1 mL), which would reduce sample consumption when sample availability is limited.

The resulting concentration data were used to characterize trace element profiles and demonstrate the applicability of the validated ICP-MS method to clinical biomonitoring investigations.

## 3. Results

### 3.1. Optimization of Sample Preparation

**Figure 1:**
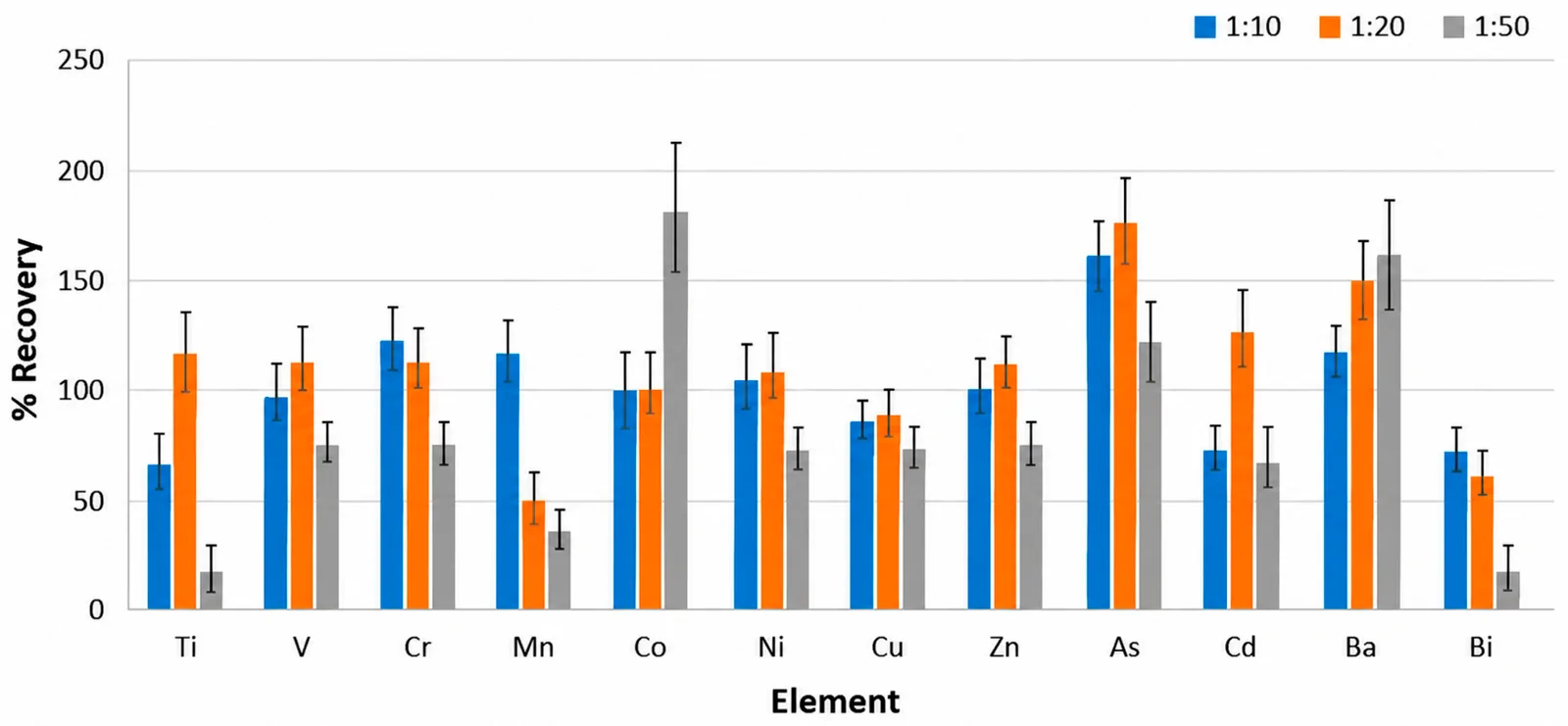
Effect of the serum dilution factor on the recovery. Error bars correspond to the confidence intervals expressed as $\pm \frac{t\cdot \;s}{\sqrt{n}}$, where t is the Student’s t value for a 95% of confidence level; s the standard deviation and n the number of replicates (n = 5). The evaluation of the different dilution factors was part of the method-development process and was considered together with the instrumental operating conditions, internal-standard correction, collision-cell conditions and rinsing procedure. The 1:10 dilution was selected for subsequent full validation because it provided the most appropriate overall balance between matrix reduction and analytical sensitivity across the complete elemental panel. The higher dilution factors were not subjected to the same full validation, as the overall analytical performance across the 12 elements was less favourable.

Sample preparation represents one of the most critical steps in the determination of trace elements in biological matrices by ICP–MS. The dilution factor was selected according to the minimum dilution that provided reliable analytical results across the complete elemental panel. Although higher dilution factors reduce the serum matrix load, they also decrease analyte concentrations in the analytical solution and may compromise the determination of elements present at low or ultratrace concentrations. This consideration was particularly relevant for elements such as arsenic, for which excessive dilution may result in concentrations approaching the analytical detection limits. Therefore, the objective was not to maximize dilution, but to identify the lowest dilution factor that provided an appropriate balance between analytical accuracy, matrix reduction and sensitivity across all investigated elements. The recoveries obtained for the three dilution factors evaluated during method development are shown in Figure 1.

The dilution experiments also provided quantitative evidence of matrix-related signal effects across the investigated elements. Increasing the dilution factor generally reduced the influence of the serum matrix, especially in the case of arsenic, but this was accompanied by a progressive loss of analytical sensitivity for several low-concentration analytes. The 1:10 dilution was therefore selected as the minimum dilution that maintained adequate sensitivity and overall analytical performance across the complete elemental panel. The subsequent recovery results obtained with certified human serum reference materials provided an additional assessment of matrix effects under the selected 1:10 conditions.

Moreover, no evidence of excessive cone contamination, signal instability or deterioration of internal standard responses was observed during routine analysis.

The selected dilution protocol also offered important practical advantages. Unlike analytical procedures based on sample digestion or wet acid mineralization, the proposed methodology required minimal sample handling, considerably reducing sample preparation time, reagent consumption and the risk of external contamination. In addition, the absence of digestion steps facilitated high-throughput analysis, making the method particularly suitable for routine clinical laboratories and large-scale biomonitoring studies.

### 3.2. Analytical Performance

External calibration using aqueous multielement standards combined with continuous correction by internal standards (^45^Sc, ^72^Ge, ^103^Rh and ^185^Re) provided excellent analytical performance for all investigated elements. Calibration curves covered the concentration interval from 0 to 5000 μg L^−1^, encompassing the concentration ranges expected in human serum while maintaining proportional instrumental response throughout the complete analytical interval. The use of aqueous standards avoided the need for matrix-matched calibration without compromising analytical accuracy, thereby simplifying routine implementation of the method.

Calibration characteristics identical to those used in the clinical study were previously established during method development (Table 2). All calibration curves showed excellent linearity, with coefficients of determination (R^2^) greater than 0.99 for every investigated element. In addition, inspection of the residual plots did not reveal any systematic deviations from linearity, confirming the suitability of the selected linear regression model over the entire working range. The slopes remained stable throughout independent calibration sequences, indicating satisfactory instrumental reproducibility.

The analytical performance of the proposed ICP–MS method was also evaluated by assessing LOD and LOQ. The corresponding validation parameters are summarized in Table 2. LOD ranged from 0.01 μg L^−1^ for V to 0.16 μg L^−1^ for Cu, whereas the corresponding LOQ ranged from 0.04 μg L^−1^ to 0.72 μg L^−1^.

To further assess the practical sensitivity of the proposed method for serum analysis, the LOQs obtained for the analytical solutions were converted to concentrations in the original serum matrix by applying the 1:10 dilution factor used during sample preparation. The resulting serum-equivalent LOQs ranged from 0.4 µg L^−1^ for V to 5.3 µg L^−1^ for Cu (Table 3).

These values were compared with serum concentrations reported for healthy individuals in an independent ICP–MS study involving a large population of healthy subjects. The comparison showed that the serum-equivalent LOQs were substantially below the median serum concentrations reported for Cu, Zn and Ba. This indicates a wide analytical margin for the determination of these elements in serum. For Ni, Mn, Cr and As, the serum-equivalent LOQs were closer to the corresponding median concentrations, whereas substantially higher LOQ-to-median ratios were observed for Co and Cd. These differences reflect the wide range of physiological concentrations encountered among trace elements and highlight the element-dependent nature of analytical sensitivity.

The comparison with published serum concentrations demonstrates that the proposed procedure provides a favourable sensitivity for several of the major and routinely determined serum trace elements, particularly Cu, Zn and Ba. For elements occurring at lower concentrations, the serum-equivalent LOQs should be interpreted with greater caution, as some physiological concentrations reported in healthy individuals fall close to or below the estimated quantification limits.

**Figure 2:**
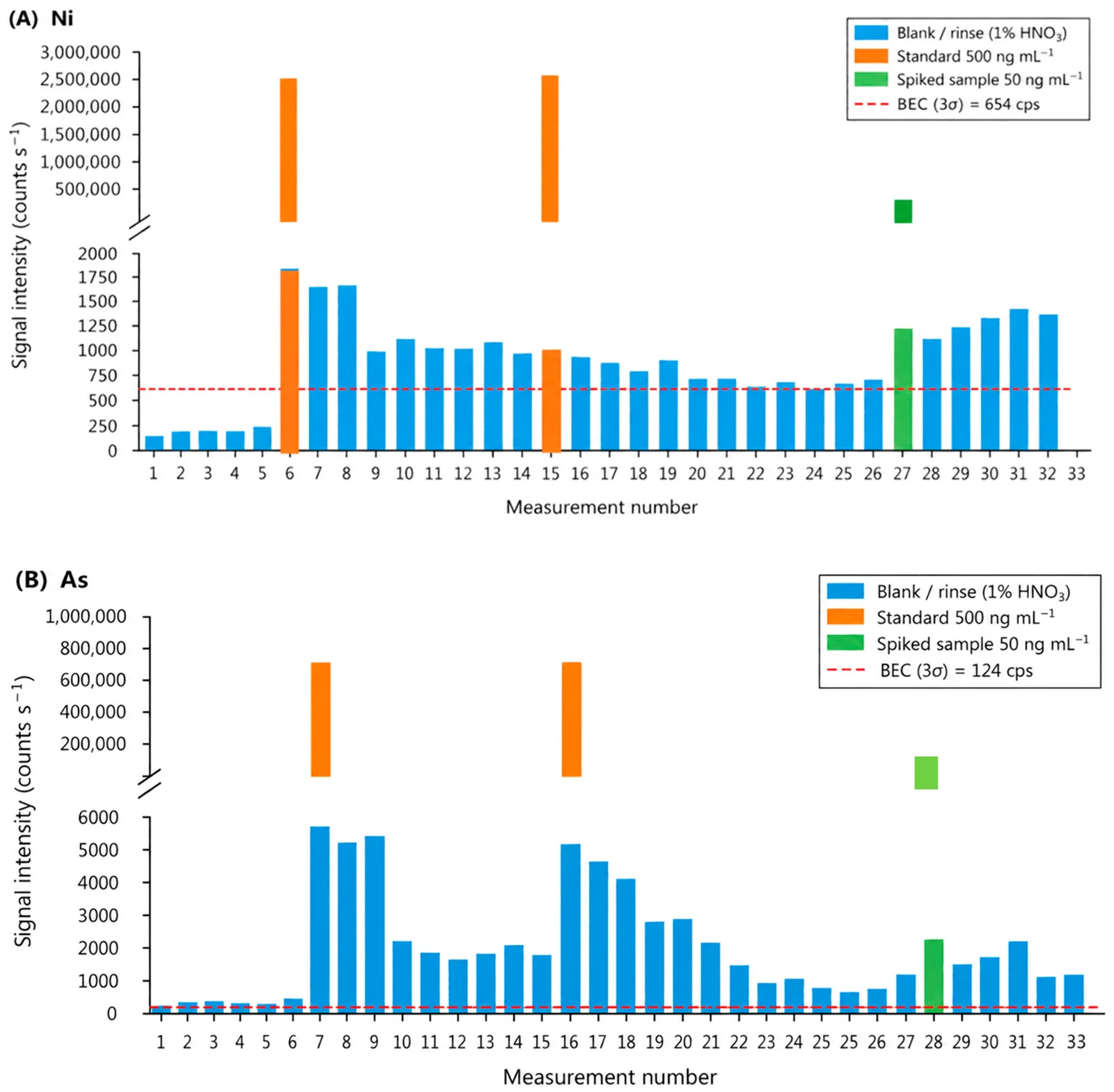
Representative blank signal evolution during successive washing cycles following the analysis of 500 ng mL^−1^ standards for (**A**) nickel, representative of analytes showing negligible carry-over, and (**B**) arsenic, representative of analytes exhibiting pronounced memory effects. Each bar corresponds to 1.5 min of washing time.

Two clearly differentiated behaviors were observed (Figure 2). Carry-over was negligible for Ti, V, Cr, Mn, Co, Ni, Cu, Zn, Cd and Ba, with approximately two rinse cycles (about 3 min) being sufficient to return the signal to baseline (<20% relative to the initial blank). In contrast, As and Bi showed more pronounced memory effects after highly concentrated solutions and required more than ten rinse cycles (approximately 15 min) to return to baseline. Accordingly, the rinsing time was adjusted to account for the element-dependent memory effects while maintaining the same rinse composition and sample introduction conditions used in the validated method.

In contrast, arsenic exhibited pronounced memory effects. Following the analysis of concentrated standards, residual signal persisted during successive blank measurements and remained clearly above baseline even after ten consecutive washing cycles (approximately 15 min). Similar behaviour was observed for Bi, although the magnitude of the effect depended on the concentration of the previously analysed solution. The results demonstrate that carry-over for these elements is concentration-dependent and may become relevant after analysing highly enriched samples.

Based on these findings, the analytical sequence incorporated additional washing cycles whenever samples with unusually high elemental concentrations were analysed. This strategy effectively minimized cross-contamination between consecutive samples while preserving analytical throughput during routine operation (see Table 4).

### 3.3. Precision Evaluation

The results for the investigated elements are summarized in Table 5 as mean concentrations, absolute confidence intervals (CI) and relative confidence intervals expressed as CI/mean (%). This parameter was used as a concentration-normalized measure of precision, allowing comparison of analytical dispersion among elements occurring at substantially different concentration levels. This approach is consistent with the use of relative measures of dispersion for characterizing analytical precision [25].

Repeatability showed relatively narrow confidence intervals for most elements. The lowest relative intervals were obtained for Cu (0.69%), As (1.67%) and Zn (2.00%). Intermediate values were observed for V (5.19%), Co (8.03%), Cr (9.70%), Ni (11.19%), Mn (11.53%), Bi (11.9%) and Ba (14.5%).

The intermediate-precision assessment showed a generally similar pattern, although the relative confidence intervals increased for some elements. The lowest values were observed for Zn (1.32%), Cu (1.90%) and As (4.12%), while V (5.79%) also showed relatively narrow intervals. Intermediate precision was broader for Ba (8.97%), Mn (9.94%), Ni (15.3%) and Co (19.9%). The largest relative intervals were observed for Cr (30.3%), Cd (79.1%) and Bi (81.1%).

The precision results showed that the magnitude of the relative confidence interval varied substantially among elements, with the narrowest intervals generally observed for elements present at higher concentrations and wider intervals for several elements occurring at lower concentrations. The results obtained under intermediate-precision conditions were generally comparable to those obtained for repeatability, although larger relative intervals were observed for Cr, Cd and Bi.

### 3.4. Trueness Evaluation

The corresponding recoveries for the investigated elements are summarized in Table 6. For most elements, the agreement between the measured and certified concentrations was broadly consistent across the two concentration levels and between short- and long-term measurements.

Cu, Zn, As and Cd showed the most consistent recoveries, generally remaining close to the certified values across the four experimental conditions. V, Cr, Co and Ni also showed recoveries close to the expected values in at least one of the concentration levels, although greater deviations were observed for some conditions. Ba showed a consistent negative bias, with recoveries of approximately 67–70% across both concentration levels and measurement periods.

More pronounced deviations were observed for Bi at Level I, while its recovery approached 100% at Level II.

The agreement between the experimentally determined and certified concentrations was further assessed by calculating the difference between both values (Δ_m_) and the corresponding uncertainty (u_Δ_) for each element, reference-material level and validation period (Table 7). In most of the evaluated conditions, the observed differences were lower than twice their associated standard uncertainties, indicating compatibility between the experimental and certified values according to the predefined criterion.

Some element and condition-specific deviations were nevertheless identified. For V, the measured difference exceeded the associated standard uncertainty under short-term Level I conditions; however, the difference remained below twice the associated uncertainty and therefore fulfilled the predefined trueness criterion. For the remaining elements, the differences between the experimental and certified concentrations were also compatible with the predefined criterion across the evaluated conditions.

### 3.5. Uncertainty Evaluation

The expanded uncertainties obtained for the investigated elements are summarized in Table 8. The trueness component included in the uncertainty budget represents the standard uncertainty associated with the comparison between the measured and certified values and should therefore not be interpreted as the analytical bias or recovery. The latter are reported separately in Table 6 and Table 7. The individual uncertainty components were expressed as relative standard uncertainties and combined by the root-sum-of-squares approach. The combined standard uncertainty was then multiplied by a coverage factor of k = 2 to obtain the expanded uncertainty reported in Table 8.

In general terms, the proposed method exhibited satisfactory uncertainty values throughout the analytical concentration range, confirming that the combined influence of the different uncertainty sources remained compatible with the analytical requirements for serum trace element determination.

As expected, repeatability represented the largest contribution to the combined uncertainty budget for most analytes, whereas the uncertainty associated with calibration contributed only marginally owing to the excellent linearity and reproducibility of the calibration curves. The uncertainty contribution associated with trueness was comparatively small for most analytes. This contribution represents the uncertainty assigned to the comparison with the certified reference materials and is therefore conceptually distinct from the recovery values and analytical differences reported in Table 6 and Table 7.

The relative contribution of each uncertainty component differed among the investigated elements. Elements routinely present at comparatively high serum concentrations, such as zinc and copper, exhibited the lowest expanded uncertainties, reflecting their excellent analytical precision and favourable signal-to-noise ratio. In contrast, cobalt, nickel and chromium showed comparatively higher uncertainty values because these elements are typically present at ultratrace concentrations, where counting statistics, instrumental background and residual spectral interferences exert a proportionally greater influence on the analytical result. Nevertheless, the calculated expanded uncertainties remained compatible with the quality specifications required for clinical trace element analysis.

The uncertainty budget also provides useful information regarding opportunities for future methodological improvement. Since repeatability constituted the dominant contribution for most analytes, further reductions in overall uncertainty should primarily focus on improving short-term measurement precision rather than modifying the calibration strategy. This finding confirms the robustness of the selected calibration model and indicates that instrumental variability represents the principal limiting factor of the analytical method.

### 3.6. Stability of the Analytes

Analyte stability was evaluated by analysing a pooled serum sample on six occasions over an eight-month period. During this period, the samples were subjected to freezing and thawing, allowing the effect of prolonged storage and repeated eight freeze–thaw exposure on the measured concentrations to be assessed. For the elements present at higher concentrations, no major changes in concentration were observed throughout the study period. However, both Cu and Zn showed a tendency towards decreasing concentrations with increasing storage time, suggesting a slight loss of these elements from the dissolved fraction during prolonged storage. In contrast, the short-term stability of both analytes was considered excellent.

The stability results indicated that prolonged storage combined with repeated freezing and thawing may lead to a reduction in the dissolved fraction of some trace elements. Nevertheless, the results supported good stability over the short- and medium-term, with the thesis specifically indicating that dissolved metal species remained stable for periods of up to approximately three months. Accordingly, repeated freeze–thaw cycles should be avoided when serum samples are intended for trace-element determination.

### 3.7. Comparison with Previously Published Methods

Compared with previously published multielement ICP–MS procedures, the present method combines direct serum dilution (1:10 with 1% HNO_3_) with simultaneous determination of 12 elements and comprehensive analytical validation. Unlike several published methods based on acid or microwave digestion, the proposed procedure requires minimal sample handling and only 0.2 mL of serum. The analytical performance is element-dependent, and although some published methods provide lower detection limits for selected analytes, the present approach offers a practical balance between analytical performance and operational simplicity. The simplified workflow may facilitate routine analysis by reducing sample preparation steps, reagent consumption and potential contamination sources. However, throughput and analytical cost were not directly quantified in the present study. Representative studies describing multielement analysis in serum using quadrupole ICP–MS are summarized in Table 9.

Regarding sample throughput, the complete workflow, including serum dilution and ICP-MS analysis, allows approximately 20 samples to be processed per hour. This throughput, together with the low serum volume required (0.2 mL) and the absence of a digestion step, supports the practical applicability of the method for routine analysis of relatively large sample series.

This method is also supported by the quality-control strategy implemented during the clinical application. Reagent blanks were analysed at regular intervals, certified serum control materials were included throughout the analytical batches, and clinical samples were analysed in triplicate. In addition, no significant instrumental drift or deterioration of calibration performance was observed during the clinical study, while internal-standard responses remained within the predefined acceptance criteria. These measures support the suitability of the validated procedure for routine clinical biomonitoring.

Another important characteristic is the use of external aqueous calibration combined with online internal standard correction. Matrix-matched calibration and standard addition procedures have frequently been recommended for biological matrices because of their potential to compensate for matrix-induced signal suppression. However, the excellent linearity, satisfactory recoveries and low analytical bias obtained during validation demonstrate that these additional calibration strategies are unnecessary under the proposed operating conditions. Consequently, the analytical workflow becomes substantially simpler and more suitable for routine implementation.

A further distinguishing feature of the present study is the comprehensive metrological characterization of the analytical method. Although most published ICP–MS validation studies report linearity, detection limits, precision and recovery, quantitative estimation of measurement uncertainty is seldom included. In the present work, uncertainty was evaluated according to the EURACHEM/CITAC and GUM recommendations, providing an objective estimate of the confidence associated with each reported concentration. This information is particularly relevant for laboratories operating under accredited quality management systems and facilitates interlaboratory comparison of analytical results.

The comparison with previously published methodologies (Table 9) indicates that the analytical performance of the present method is within the range reported for multielement ICP–MS determination in biological matrices. The LODs obtained in the present study (0.01–0.16 µg L^−1^) are comparable to those reported for several previously validated methods, although some studies achieved lower detection limits for selected elements, particularly when acid digestion or more extensive sample preparation procedures were employed. Conversely, the present method combines these analytical characteristics with a simple 1:10 direct-dilution procedure, avoiding digestion and substantially reducing sample manipulation, reagent consumption and potential sources of contamination. This balance between analytical performance and operational simplicity is particularly relevant for routine analysis of large numbers of serum samples.

### 3.8. Application of the Analysis Method to Human Serum Samples

Following analytical validation, the proposed ICP–MS method was applied to the determination of trace elements in serum samples obtained from healthy volunteers and patients undergoing chronic haemodialysis. Sample preparation, instrumental conditions and quality control procedures were identical to those employed during method validation, thereby ensuring full consistency between validation and routine application.

The analytical performance observed during validation was maintained throughout the clinical study. No significant instrumental drift, carry-over or deterioration of calibration performance was detected during the analytical sequence, and internal standard recoveries remained within the predefined acceptance limits for all analysed samples. The simplicity of the direct-dilution procedure enabled rapid processing of a large number of clinical specimens while minimizing sample handling and the associated risk of external contamination.

The obtained analytical performance allowed the identification of differences in serum trace element profiles between both populations, demonstrating that the method is suitable for clinical biomonitoring applications involving both essential and potentially toxic trace elements. The biological interpretation of these findings has been reported separately.

The serum concentrations of the elements analysed in the study are summarised in Table 10. It was observed that the distribution of several elements differed between healthy controls and patients undergoing hemodialysis.

Patients undergoing hemodialysis showed higher serum concentrations of Cr, As, Ni and Co compared with the control group. The differences were particularly marked for chromium and nickel. The interquartile range for Cr was 3.51–9.68 µg L^−1^ in haemodialysis patients compared with 0.02–1.34 µg L^−1^ in controls, whereas for Ni the corresponding ranges were 2.45–6.63 µg L^−1^ and 0.02–0.50 µg L^−1^, respectively. Arsenic also showed higher concentrations in hemodialysis patients, with interquartile ranges of 4.11–19.09 µg L^−1^ compared with 0.99–5.53 µg L^−1^ in controls. Similarly, higher concentrations were observed for Co (0.37–0.76 vs. 0.19–0.38 µg L^−1^).

In contrast, Zn showed lower serum concentrations in patients undergoing hemodialysis. The interquartile ranges for Zn were 530.50–724.10 µg L^−1^ in hemodialysis patients and 724.30–861.30 µg L^−1^ in controls.

For V, Cu, Mn and Ba, the distributions showed greater overlap between the two groups. The interquartile ranges were 0.89–1.47 vs. 0.47–1.31 µg L^−1^ for V, 845.20–1092.50 vs. 834.60–1132.60 µg L^−1^ for Cu, 3.24–7.16 vs. 3.35–7.09 µg L^−1^ for Mn, and 3.01–8.53 vs. 3.48–7.59 µg L^−1^ for Ba in hemodialysis patients and controls, respectively.

From an analytical perspective, most elements had no values below the limit of detection. However, Ni showed 25% of measurements below the LOD, Cr 13%, As and V 5%, and Ba 0.5%. In addition, missing values were present for some elements, particularly Ti and Co (10%), resulting in reduced effective sample sizes for these analytes. Measurements below the LOD were considered non-detectable and were not included as quantitative values in the statistical analyses. Measurements between the LOD and the LOQ were retained and reported as quantified results, although they were interpreted with caution.

Finally, the successful application of the validated procedure to a clinical cohort confirms its robustness under routine laboratory conditions and supports its use for multielement biomonitoring studies requiring reliable determination of trace elements in human serum.

## 4. Discussion

The selection of an appropriate sample preparation procedure is a critical step in ICP–MS analysis of serum because proteins, lipids and dissolved salts can affect aerosol generation, plasma stability and ion transmission. In the present study, direct dilution with 1% (*v*/*v*) HNO_3_ provided an appropriate compromise between matrix reduction and analytical sensitivity. A 1:10 dilution was selected following comparison of different dilution factors, as higher dilution reduced the signal for several low-concentration analytes. Compared with digestion-based procedures, direct dilution simplifies sample preparation, reduces reagent consumption and analysis time, and limits potential contamination, while providing satisfactory analytical performance when combined with instrumental optimization and internal-standard correction.

The 1:10 dilution also results in a higher residual serum matrix than methods using greater dilution factors. This was considered an inherent trade-off of the selected approach, since increasing the dilution factor would reduce matrix loading but also decrease the concentration of low-abundance analytes. Consequently, the use of a 1:10 dilution may require particular attention to rinsing during long analytical sequences, especially when samples with high analyte concentrations are analyzed. In the present method, this aspect was addressed by optimizing the rinsing time under high-concentration conditions, while maintaining the same rinse composition and sample introduction conditions used for validation.

The method showed good overall analytical performance, with calibration coefficients of determination above 0.99 across the investigated ranges and no relevant systematic deviations from linearity. The analytical sensitivity was, however, element-dependent. Comparison of the serum-equivalent LOQs with reported physiological concentrations showed a wide variation in the analytical margin available for individual elements. The method therefore provides particularly favourable sensitivity for elements occurring at relatively high serum concentrations, whereas the determination of elements present at ultratrace levels requires greater caution. This distinction is important when assessing fitness for purpose, since an analytical method should be judged according to the concentration range and biological question relevant to its intended application rather than by its absolute LOQ alone.

The serum concentrations obtained in the clinical application were generally within the concentration ranges reported for healthy populations for the elements for which established reference data were available. However, the present study was not designed to establish biological reference intervals, and the reported ranges should therefore not be interpreted as laboratory reference intervals for clinical decision-making. Similarly, although the analytical performance was considered adequate for the intended biomonitoring application, clinical decision limits and fitness for individual clinical decision-making were beyond the scope of the present validation study.

The precision results were also element-dependent. Relative confidence intervals were generally narrower for elements present at higher serum concentrations and wider for several low-abundance elements. Importantly, intermediate precision remained broadly comparable with repeatability for most analytes, indicating that extending the measurements across different analytical days did not introduce a major additional source of variability. The wider relative intervals observed for some low-concentration elements should therefore be interpreted together with their absolute concentrations, LOQs and measurement uncertainties rather than considered in isolation. Internal-standard correction was retained in the final procedure as a means of controlling instrumental drift and residual matrix effects, even though its effect on precision was not uniform across all analytes. The trueness results further supported the adequacy of this approach. Thus, for instance, arsenic showed recoveries between 113% and 117% across both concentration levels and validation periods (Table 6), indicating no relevant systematic bias under the selected analytical conditions.

Despite this element-dependent variability, several elements of particular relevance for serum biomonitoring showed satisfactory analytical performance under the selected conditions. Copper and zinc showed recoveries close to the certified values (91–96% and 85–93%, respectively), together with low intermediate precision (1.90% and 1.32%). Arsenic showed a moderate positive deviation in recovery (111–117%), but the corresponding differences between measured and certified values remained compatible with the predefined trueness criterion. Manganese, nickel and cadmium also fulfilled the predefined trueness criterion, although with greater variability for some validation parameters. These results support the applicability of the method for selected trace elements in serum, while highlighting the need for element-specific assessment of analytical performance.

The trueness assessment provided generally satisfactory agreement with the certified serum reference values across two concentration levels and both short- and long-term conditions. Cu, Zn, As and Cd showed the most consistent agreement, while most of the remaining analytes were also compatible with their certified values when the associated uncertainties were considered. Ba illustrates the importance of evaluating trueness together with uncertainty, as its recoveries of approximately 67–70% did not correspond to statistically significant differences from the certified values when the associated uncertainties were taken into account.

Measurement uncertainty was incorporated as an integral component of the validation. The uncertainty budgets included calibration, repeatability and trueness contributions, with repeatability representing the predominant contribution for most analytes. This metrological characterization provides a quantitative estimate of confidence in the reported concentrations and strengthens the suitability of the method for applications requiring traceable analytical results and documented measurement performance.

The stability study demonstrated that pre-analytical handling may represent an additional source of variability. Most analytes remained relatively stable during the evaluated period, although Cu, Zn, Co and As showed evidence of concentration changes during prolonged storage involving repeated freeze–thaw cycles. These findings support the use of standardized storage conditions and minimization of repeated freezing and thawing, particularly when samples are intended for retrospective or long-term biomonitoring studies.

Compared with previously reported serum ICP–MS methods, the proposed procedure provides a comparable analytical performance while requiring a simpler sample preparation strategy. The combination of direct 1:10 dilution, quadrupole ICP–MS with helium collision-cell technology, multielement determination and comprehensive metrological characterization represents a practical balance between analytical performance and operational simplicity. The inclusion of measurement uncertainty and systematic assessment of trueness and stability further strengthens the validation beyond the conventional reporting of calibration, precision and detection limits.

The application to real serum samples demonstrated that the method can accommodate the broad concentration ranges encountered in healthy individuals and patients undergoing chronic hemodialysis. Marked differences were observed for several elements, including higher concentrations of Cr, Ni, As, Co and Ti and lower concentrations of Zn in the hemodialysis group, whereas other elements showed greater overlap between groups. These findings demonstrate the practical applicability of the method to clinical serum samples containing elements over markedly different concentration ranges. The detailed biological and clinical interpretation of these differences is beyond the scope of the present analytical study and has been addressed separately. It should be emphasized that the present study represents an analytical method validation and an assessment of applicability to human serum samples, rather than a clinical validation study. The successful measurement of clinical samples demonstrates the practical applicability of the method but does not establish diagnostic accuracy, clinical sensitivity or specificity, or clinical decision-making performance.

The differences observed between healthy controls and patients undergoing hemodialysis should be interpreted in the context of the multiple factors that may influence trace-element homeostasis in this population. Renal dysfunction, dialysis treatment, dietary restrictions, chronic inflammation, medication use, nutritional status and supplementation, as well as exposure to dialysis equipment and fluids, may all contribute to alterations in serum trace-element concentrations. Therefore, the observed differences cannot be attributed solely to the dialysis procedure itself. In the present study, the clinical application was primarily intended to demonstrate the applicability of the analytical method, and detailed investigation of the individual contribution of these potential confounding factors was beyond the scope of the study.

## 5. Strengths and Limitations

The present study has several strengths. First, the proposed ICP–MS procedure combines a simple sample preparation strategy based on direct serum dilution with a comprehensive analytical validation covering calibration performance, analytical sensitivity, precision, trueness, measurement uncertainty and analyte stability. The use of a 1:10 dilution minimizes sample handling and reagent consumption while providing an appropriate balance between matrix reduction and analytical sensitivity. Second, the validation included a broad multielement panel, allowing essential and potentially toxic trace elements to be determined within a single analytical procedure. The evaluation of trueness at two concentration levels and under both short- and long-term conditions, together with the inclusion of measurement uncertainty, provides a more comprehensive characterization of method performance than reliance on calibration and precision parameters alone. Finally, the application to real serum samples from healthy individuals and patients undergoing chronic hemodialysis demonstrated the practical applicability of the method across markedly different concentration ranges.

Several limitations should nevertheless be considered. The method was validated on a single quadrupole ICP–MS platform and within a single laboratory; therefore, its transferability to other instruments, laboratories and operating conditions remains to be established through interlaboratory studies. The stability assessment indicated that prolonged storage combined with repeated freeze–thaw cycles may affect the measured concentrations of some analytes, highlighting the importance of standardized pre-analytical handling. Finally, the clinical application was intended primarily to demonstrate the applicability of the analytical procedure rather than to provide clinical validation or establish diagnostic performance. Successful measurement of serum samples does not, by itself, establish clinical sensitivity, specificity, diagnostic accuracy or clinical decision limits. Although several clinical and dialysis-related variables were recorded, the study was not specifically designed to determine the individual contribution of renal dysfunction, dialysis treatment, medication, nutritional status, supplementation, or exposure to dialysis equipment and fluids to the observed elemental concentrations. Therefore, the observed differences should not be interpreted as being attributable solely to hemodialysis.

Potential pre-analytical contamination and analyte losses associated with collection and storage materials were minimized by using trace-element serum collection tubes and acid-decontaminated polypropylene microtubes. Nevertheless, adsorption of trace elements to tube surfaces cannot be completely excluded and should be considered as a potential pre-analytical source of variability, particularly at low analyte concentrations.

## 6. Conclusions

The present study demonstrates that robust multielement analysis of human serum does not necessarily require complex sample preparation or highly sophisticated analytical workflows. By combining a simple direct-dilution protocol with comprehensive analytical validation and metrological evaluation, the proposed ICP–MS methodology provides a practical and reliable approach for the routine determination of essential and potentially toxic trace elements in clinical laboratories.

Beyond the analytical performance itself, one of the principal contributions of this work is the integration of measurement uncertainty into the validation strategy. Although uncertainty estimation is recognised as a fundamental component of analytical quality, it remains insufficiently addressed in many published ICP–MS methodologies for biological matrices. Incorporating this metrological perspective strengthens the reliability, traceability and comparability of analytical results, facilitating their implementation in laboratories operating under accredited quality management systems.

The proposed validation strategy may therefore serve not only as a framework for serum trace element analysis but also as a methodological model for the development and validation of ICP–MS procedures applied to other biological matrices. As interest in trace element profiling continues to expand in clinical chemistry, nutritional research, toxicology and human biomonitoring, analytical methods should aim not only for high sensitivity but also for demonstrable robustness and metrological transparency.

## Figures and Tables

**Table 1:** ICP-MS operating conditions used for the determination of trace elements in human serum.

Parameter	Value
RF power	1550 W
Plasma gas flow rate (Ar)	15.0 L min^−1^
Auxiliary gas flow rate (Ar)	0.90 L min^−1^
Carrier gas flow rate (Ar)	1.05 L min^−1^
Collision gas	Helium
Collision gas flow rate	4.5 mL min^−1^
Nebulizer type	MicroMist concentric nebulizer
Spray chamber	Quartz Scott-type spray chamber
Sample uptake rate	0.40 mL min^−1^
Data acquisition mode	Spectrum mode
Replicates per sample	3
Dwell time	20 ms
Settling time	40 ms
Internal standards	^45^Sc, ^72^Ge, ^103^Rh and ^185^Re (50 ng mL^−1^ of each)
Collision cell mode	Helium mode (4.3 mL min^−1^)
Isotopes monitored	^47^Ti, ^51^V, ^52^Cr, ^55^Mn, ^59^Co, ^60^Ni, ^63^Cu, ^66^Zn, ^75^As, ^111^Cd, ^137^Ba, ^209^Bi

**Table 2:** Analytical performance of the ICP–MS method.

Isotope	Calibration Range (µg L^−1^)	R^2^	LOD (µg L^−1^)	LOQ (µg L^−1^)
^48^Ti	0.510	1.0000	0.05	0.17
^51^V	0.5–10	0.9999	0.01	0.04
^52^Cr	0.5–10	0.9999	0.04	0.12
^55^Mn	0.5–10	1.0000	0.07	0.24
^59^Co	0.5–10	0.9999	0.02	0.06
^60^Ni	0.5–10	0.9999	0.04	0.14
^63^Cu	1.0–500	0.9999	0.16	0.53
^66^Zn	1.0–500	1.0000	0.15	0.50
^75^As	0.5–10	1.0000	0.02	0.07
^111^Cd	0.5–10	0.9999	0.03	0.09
^137^Ba	1.0–50	0.9999	0.06	0.19
^209^Bi	0.5–10	0.9993	0.05	0.16

**Table 3:** Comparison of serum-equivalent limits of quantification with reported serum concentrations in healthy individuals ^&^.

Element	Serum-Equivalent LOQ (µg L^−1^) ^@^	Healthy Serum, P5–P95 (µg L^−1^)	LOQ/Median Serum Concentration
Ti	1.70	n.a.	n.a.
V	0.40	0.180–0.543 *	1.39 *
Cr	1.20	0.488–3.067 *	1.28 *
Mn	2.40	1.718–4.357 *	0.98 *
Co	0.60	0.070–0.362 *	5.66 *
Ni	1.40	1.397–8.066 *	0.57 *
Cu	5.30	652–1253 *	0.006 *
Zn	5.00	649–1080 *	0.006 *
As	0.70	0.335–1.485 *	1.21 *
Cd	0.90	0.025–0.139 *	17.31 *
Ba	1.90	14.894–50.303 *	0.07 *
Bi	1.60	<0.007–0.067 #	n.a #

^&^ P5–P95: 5th and 95th percentiles of the serum concentration distribution reported in healthy individuals; LOQ-to-median ratio: ratio between the serum-equivalent LOQ of the present method and the median serum concentration (P50) reported in the reference population. Serum-equivalent LOQs: were calculated by multiplying the LOQ obtained for the analytical solution by the sample dilution factor (10). n.a.: Not available. ^@^ Serum equivalent LOQ, defined as the calculated LOQ × dilution factor (i.e., 10). * Data taken from ref. [23]. # Data taken from ref. [24].

**Table 4:** Summary of carry-over behaviour observed for the investigated trace elements.

Behaviour	Elements	Washing Required
Negligible carry-over	Ti, V, Cr, Mn, Co, Ni, Cu, Zn, Cd, Ba	2 cycles (~3 min)
Pronounced memory effects	As, Bi	>10 cycles (~15 min) after highly concentrated samples

**Table 5:** Repeatability and intermediate precision of the validated ICP–MS method *.

Element	Repeatability			Intermediate Precision		
	Mean (µg L^−1^)	CI (µg L^−1^)	CI/mean (%)	Mean (µg L^−1^)	CI (µg L^−1^)	CI/mean (%)
Ti #	n.a.	n.a.	n.a.	n.a.	n.a.	n.a.
V	0.89	±0.05	5.19	0.95	±0.05	5.79
Cr	0.91	±0.09	9.70	2.29	±0.69	30.3
Mn	3.74	±0.43	11.53	2.37	±0.24	9.94
Co	0.36	±0.03	8.03	0.34	±0.067	19.9
Ni	1.41	±0.16	11.19	1.86	±0.28	15.3
Cu	1116	±7.67	0.69	1193	±22.7	1.90
Zn	958	±19.1	2.00	924	±12.2	1.32
As	6.56	±0.11	1.67	4.02	±0.16	4.12
Cd	0.27	±0.02	7.40	0.03	±0.02	79.1
Ba	2.83	±0.41	14.5	2.84	±0.25	8.97
Bi	0.59	±0.07	11.9	0.91	±0.73	81.1

* Data obtained from the pooled serum sample. 95% Confidence intervals calculated as: CI = ±$\frac{t\;\cdot \;s}{\sqrt{n}}$, where t was the Student’s t for a 95% confidence level, s the standard deviation and n the number of replicates. n.a.: Not available. # Ti was not detected in the pooled sample.

**Table 6:** Trueness of the ICP–MS method evaluated using certified serum reference materials at two concentration levels and under short- and long-term conditions *.

Element	Short-Term Level I (%)	Short-Term Level II (%)	Long-Term Level I (%)	Long-Term Level II (%)
Ti	n.a.	137	n.a.	n.a.
V	55	99	65	104
Cr	144	106	151	109
Mn	n.a.	109	n.a.	113
Co	119	108	174	81.1
Ni	129	85	137	104
Cu	92	91	96	94
Zn	93	87	87	85
As	113	117	113	111
Cd	89	89	83	91
Ba	68	67	70	69
Bi	206	110	182	101

* n.a.: Not available.

**Table 7:** Differences between measured and certified concentrations (Δ_m_) and associated uncertainties (u_Δ_) for the assessment of trueness *.

Element	Short-Term L-I Δ_m_/u_Δ_	Short-Term L-II Δ_m_/u_Δ_	Long-Term L-I Δ_m_/u_Δ_	Long-Term L-II Δ_m_/u_Δ_
Ti	n.a.	39/111	n.a.	n.a.
V	1.1/1.0	0.1/4.4	0.9/1.1	0.4/4.4
Cr	1.7/5.7	0.5/3.7	2.0/2.1	0.8/3.3
Mn	0.8/4.2	1.2/4.3	—	—
Co	0.3/0.8	0.3/1.4	1.3/2.1	0.6/2.1
Ni	1.2/2.1	1.3/3.9	1.5/2.4	0.4/5.0
Cu	67/241	117/430	34/267	82/441
Zn	97/401	259/732	167/439	300/671
As	1.3/4.3	3.3/8.8	1.3/4.8	2.1/9.0
Cd	0.2/0.9	0.5/2.4	0.4/1.0	0.4/2.0
Ba	30/38	50/65	28/45	47/67
Bi	1.0/0.8	0.4/1.9	0.8/2.3	0.05/5.1

* Δ_m_, difference between the experimentally measured and certified concentrations; u_Δ_, uncertainty associated with the difference. Values are presented as Δ_m_/u_Δ_. L-I and L-II, certified serum reference material Levels I and II, respectively; short-term and long-term conditions correspond to the respective validation periods. n.a.: Not available.

**Table 8:** Measurement uncertainty budget for the validated ICP–MS method *.

Element	Calibration (%)	Repeatability (%)	Uncertainty Contribution from Trueness (%)	Combined Uncertainty (%)	Expanded Uncertainty (%)
Ti	n.a.	n.a.	n.a.	n.a.	n.a.
V	0.113	2.482	0.45	2.525	5.05
Cr	0.112	2.475	0.37	2.505	5.01
Mn	0.116	5.873	0.65	5.910	11.82
Co	0.129	2.020	0.71	2.145	4.29
Ni	0.166	7.811	0.59	7.835	15.67
Cu	0.124	2.116	0.36	2.150	4.30
Zn	0.086	2.026	0.39	2.065	4.13
As	0.070	3.513	0.47	3.545	7.09
Cd	0.185	5.911	0.50	5.935	11.87
Ba	0.117	1.061	0.66	1.255	2.51
Bi	≈0.000	0.813	1.04	1.320	2.64

* Combined standard uncertainty was calculated according to the EURACHEM/CITAC approach. Expanded uncertainty was obtained using a coverage factor of k = 2. n.a.: Not available.

**Table 9:** Comparison of the proposed ICP–MS method with representative methodologies for multielement determination in human serum *.

Matrix	Sample Preparation	No. of Elements	Sample Volume	LOD/LOQ (µg L^−1^)	Precision	Recovery/Accuracy	Validation	Ref.
Serum/Whole blood	Acid digestion	10 in serum	100 µL	Serum LOD: 0.01–3.96 (Au, Cu, Se, Zn, Fe, Mg, Mn, Cr, Ni, Li)/LOQ: 0.03–7.93	<15% CV for most elements	80–130% for most elements	Linearity, LOD, LOQ, precision, accuracy, EQA	[26]
Human serum	Acid digestion	25	20 µL	LOD: 0.0015–11.6500 (U–Li)/LOQ: lowest non-zero calibration standard	<15% CV (intra- and inter-day)	Recovery/accuracy evaluated; recoveries generally > 90%	Linearity, LOD, recovery, accuracy, precision	[27]
Serum/Whole blood	Microwave acid digestion	8 minerals in serum	250 µL	Not reported as LOD/LOQ	0.635–10.1% RSD (serum)	Not reported	Linearity, intra-/interday precision and other figures of merit	[28]
Human serum	Acid digestion/dilution	14	Not specified	Not reported	Not reported as a validation precision study	Reference values/analytical characterization	Reference values and analytical validation	[29]
Plasma/Serum	Direct dilution	8	150 µL	0.019–8.6 (Co, Mn, Cd, Pb, Cu, Mg, Fe, Zn)/—	7.5% Mn; 16% Co; 131% Cd; 8.0% Pb; 8.4% Mg; 9.4% Fe; 8.3% Cu; 9.9% Zn	76–10 0%	Linearity, LOD, accuracy, precision, QC	[30]
Human serum	Direct dilution (1:10 with 1% HNO_3_)	12	200 µL	0.01–0.16/0.04–0.53	1.32–81.1% relative confidence Interval (CI/mean)	55–206%	Complete validation (linearity, LOD, LOQ, carry-over, precision, trueness, stability and measurement uncertainty)	This work

* EQA, external quality assessment; QC, quality control; CV, coefficient of variation; CI, confidence interval.

**Table 10:** Serum concentrations of trace elements in healthy controls and patients undergoing chronic hemodialysis *.

Element	Controls (µg L^−1^)	Hemodialysis Patients (µg L^−1^)
Ti	21–28	23–34
V	0.47–1.31	0.89–1.47
Cu	834.60–1132.60	845.20–1092.50
Zn	724.30–861.30	530.50–724.10
Mn	3.35–7.09	3.24–7.16
Ba	3.48–7.59	3.01–8.53
Cr	0.02–1.34	3.51–9.68
As	0.99–5.53	4.11–19.09
Ni	0.02–0.50	2.45–6.63
Co	0.19–0.38	0.37–0.76
Cd	n.d.	n.d.
Bi	n.d.	n.d.

* Values are expressed as median [interquartile range (IQR)]. IQR corresponds to the 25th–75th percentile range. n.d.: Not detected.

## Data Availability

The data presented in this study are available on request from the corresponding author due to privacy and ethical restrictions.
